# PRRSV-1 induced lung lesion is associated with an imbalance between costimulatory and coinhibitory immune checkpoints

**DOI:** 10.3389/fmicb.2022.1007523

**Published:** 2023-01-12

**Authors:** Inés Ruedas-Torres, José María Sánchez-Carvajal, Librado Carrasco, Francisco José Pallarés, Fernanda Larenas-Muñoz, Irene Magdalena Rodríguez-Gómez, Jaime Gómez-Laguna

**Affiliations:** Department of Anatomy and Comparative Pathology and Toxicology, Pathology and Immunology Group (UCO-PIG), UIC Zoonosis y Enfermedades Emergentes ENZOEM, International Agrifood Campus of Excellence (ceiA3), Faculty of Veterinary Medicine, University of Córdoba, Córdoba, Spain

**Keywords:** immune checkpoints, dysregulation, lung, lymph node, PRRSV, virulence

## Abstract

*Porcine reproductive and respiratory syndrome virus* (PRRSV) induces a dysregulation on the innate and adaptive immune responses. T-cell activation requires a proper interaction and precise balance between costimulatory and coinhibitory molecules, commonly known as immune checkpoints. This study aims to evaluate the expression of immune checkpoints in lung and tracheobronchial lymph node from piglets infected with two PRRSV-1 strains of different virulence during the early stage of infection. Seventy 4-week-old piglets were grouped into three experimental groups: (i) control, (ii) 3249-infected group (low virulent strain), and (iii) Lena-infected group (virulent strain) and were euthanized at 1, 3, 6, 8, and 13 days post-infection (dpi). Lung and tracheobronchial lymph node were collected to evaluate histopathological findings, PRRSV viral load and mRNA expression of costimulatory (*CD28*, *CD226*, *TNFRSF9*, *SELL*, *ICOS*, and *CD40*) and coinhibitory (*CTLA4*, *TIGIT*, *PD1/PDL1*, *TIM3*, *LAG3*, and *IDO1*) molecules through RT-qPCR. Our findings highlight a mild increase of costimulatory molecules together with an earlier and stronger up-regulation of coinhibitory molecules in both organs from PRRSV-1-infected animals, especially in the lung from virulent Lena-infected animals. The simultaneous expression of coinhibitory immune checkpoints could work in synergy to control and limit the inflammation-induced tissue damage. Further studies should be addressed to determine the role of these molecules in later stages of PRRSV infection.

## 1. Introduction

First isolated in the Netherlands in 1991, *porcine reproductive and respiratory syndrome virus* (PRRSV) is one of the most important diseases for pig production worldwide (Wensvoort et al., 1991; Holtkamp et al., 2013). PRRSV is an RNA virus with a remarkable genetic and antigenic variability classified as two different viral species, *Betaarterivirus suid-1* (or PRRSV-1) and *Betaarterivirus suid-2* (or PRRSV-2); (Brinton et al., 2018). In addition, diverse outbreaks caused by virulent PRRSV-1 and PRRSV-2 strains have been described around the world since the appearance of the disease (Tian et al., 2007; Zhou et al., 2008; Karniychuk et al., 2010; Morgan et al., 2013). Regardless of the virulence of the strain, PRRSV elicits a poor innate and adaptive immune response, supporting viral replication and persistence in the host (Butler et al., 2014; Lunney et al., 2016). However, some differences, regarding the IFN-γ expression and the frequencies of CD4^+^ T cells and CD8^+^ T cells, between infected animals with low virulent and virulent PRRSV strains have been described (Morgan et al., 2013; Weesendorp et al., 2013).

A proper T-cell activation requires three simultaneous essential signals: (i) peptide recognition by the interaction among T cell receptor (TCR) on the surface of T cells and major histocompatibility complex (MHC) on the surface of antigen presenting cells (APCs); (ii) the interaction of costimulatory molecules expressed on APCs to their counterparts on T cells; and (iii) the signaling of certain polarizing cytokines (Viganò et al., 2012; Sckisel et al., 2015). In this context, the perturbation of any of these interactions, not only by the lack of costimulatory signals but also by the expression of coinhibitory signals, may affect T-cell activation, inducing an anergic state of T cells (Korman et al., 2006; Viganò et al., 2012; Attanasio and Wherry, 2016). These costimulatory and coinhibitory molecules are commonly described as ‘immune checkpoints’ (Korman et al., 2006). Whereas positive costimulation is required for the development of a proper T-cell immune response to tackle infectious diseases, the inhibitory costimulation regulates peripheral tolerance mechanisms to reduce inflammation-induced tissue damage, acting as a ‘brake’ for the immune system (Viganò et al., 2012; Cai et al., 2020). Therefore, in the case of acute and chronic infectious diseases, immune checkpoints have been reported to contribute regulating the host’s immune response (Jiang and Chess, 2006; Viganò et al., 2012; Attanasio and Wherry, 2016; Wykes and Lewin, 2018; Cai et al., 2020).

CD28 family is one of the major receptor families of costimulatory and coinhibitory molecules involved in regulation of lymphocytes, including CD28 and inducible T-cell costimulator (ICOS) as activation signals, and cytotoxic T-lymphocyte antigen 4 (CTLA4), and programmed cell death protein 1 (PD1) as inhibitory signals (Krueger et al., 2017). In PRRSV field, Richmond et al. (2015a) analyzed the role of the ligand of PD1, programmed death-ligand 1 (PDL1), in monocyte-derived dendritic cells (MoDCs) infected with different combinations of porcine circovirus type 2 (PCV2) and PRRSV strains of different virulence. Only MoDCs infected with the virulent PRRSV-2 VR-2385 strain alone and the combination of PCV2 with all the other PRRSV-2 strains (including VR-2385, NADC-20, PRRSV-MLV) showed a significant increase in PDL1 expression (Richmond et al., 2015a). The role of PD1/PDL1 axis in lymphocyte anergy and apoptosis phenomena in pigs was demonstrated by the same authors in a parallel study (Richmond et al., 2015b). Moreover, a marked up-regulation of *PDL1* and *CTLA4* genes has been also observed in the thymus of piglets infected with the virulent PRRSV-1 Lena strain (Ruedas-Torres et al., 2021b). In this context, transcriptional genome analysis performed by our research group and others have also described an overexpression of some of these costimulatory and coinhibitory molecules in target organs from PRRSV-1 and PRRSV-2 infected pigs (Chaudhari et al., 2020, 2021; Fleming et al., 2020; Sánchez-Carvajal et al., 2021a). In this sense, Chaudhari et al. (2020) already described an up-regulation of coinhibitory molecules such as TIGIT, PD1, TIM3, and IDO1 in the inguinal lymph node from pigs infected with a live-attenuated PRRSV-2 strain. The same authors described an increase of PDL1, PDL2, IDO1, and other inhibitory immune checkpoints in PAMs from animals infected with a virulent PRRSV-2 strain (Chaudhari et al., 2021).

Despite these preliminary results, the role that immune checkpoints may play during PRRSV-1 infection is barely known, existing still numerous gaps to be addressed at this regard to unravel the modulation of the host immune response. Hence, this study aims to evaluate the expression of some costimulatory (*CD28, CD226, TNFRSF9, SELL, ICOS and CD40*) and coinhibitory (*CTLA4, TIGIT, PD1/PDL1 axis, TIM3, LAG3, and IDO1*) molecules in the lung and tracheobronchial lymph node from piglets infected with two PRRSV-1 strains of different virulence, virulent Lena strain and low virulent 3249 strain, during the early stage of infection.

## 2. Materials and methods

### 2.1. Animals and experimental design

This *in vivo* study was part of a larger project to explore the pathogenesis evoked by PRRSV-1 strains of different virulence. Animals and samples were collected from an experiment previously published (Rodríguez-Gómez et al., 2019). In brief, seventy 4-week-old Landrace × Large White piglets, negative for PRRSV (PRRS X3 Ab Test, IDEXX Laboratorios, S.L., Barcelona, Spain), *Mycoplasma hyopneumoniae* [in-house PCR against *M. hyopneumoniae* (Mattsson et al., 1995)] and PCV-2 (Sibila et al., 2004), were randomly located in three different pens at the Centre de Recerca en Sanitat Animal (IRTA-CReSA, Cerdanyola del Vallès, Barcelona, Spain). After a week of acclimatization, piglets were intranasally inoculated as follows: 16 pigs with 2 ml of porcine alveolar macrophages supernatant diluted in RPMI 1640 medium (Thermo Fisher Scientific, Barcelona, Spain; control group); 26 pigs with 2 ml of 10^5^ TCID_50_ of the low virulent PRRSV-13249 strain (subtype 1; 3249-infected group; Gimeno et al., 2011); and 28 pigs with 2 ml of 10^5^ TCID_50_ of the virulent PRRSV-1 Lena strain (subtype 3; Lena-infected group; Karniychuk et al., 2010). At 1, 3, 6 and 8 days post-infection (dpi), 3 pigs from the control group and 5 from each infected group were humanely euthanized. At 13 dpi, 4, 6 and 8 animals from control, 3249- and Lena-infected groups, respectively, were euthanized under the same conditions. Clinical signs (liveliness, respiratory signs, and anorexia) and rectal temperature were monitored from 1 day prior to inoculation throughout the study (see Rodríguez-Gómez et al., 2019 for details). During the necropsies, gross lung lesions were recorded as described elsewhere (Rodríguez-Gómez et al., 2019). Samples from cranial, middle and caudal lobes from the right lung and tracheobronchial lymph node were collected and immersed in TRIzol™ LS Reagent (Invitrogen, Carlsbad, CA, USA) and frozen at −80°C until further processing. Moreover, these tissues were fixated in 10% neutral buffered formalin and sectioned at 4 μm for the corresponding histopathological studies.

This experiment was approved by the IRTA Ethics Committee and by the Catalan Autonomous Government (Project 3,647; FUE-2017-00533413) and carried out following the European Union guidelines (Directive 2010/63/EU).

### 2.2. Gross and histopathological examination

For gross examination of the lung, a percentage reflecting the approximate volume of the affected lung parenchyma with respect to the entire lung was assigned to each lung lobe. The sum of all frequencies was an estimation of the percentage of the affected lung in a scale from 0 to 100 (Rodríguez-Gómez et al., 2019). Microscopic evaluation of lung and tracheobronchial lymph node lesion was conducted as previously described (Rodríguez-Gómez et al., 2019; Ruedas-Torres et al., 2021a). Hematoxylin and eosin-stained sections from cranial, middle and caudal lobes from the right lung and tracheobronchial lymph node were blindly examined and scored by two different pathologists. Briefly, in each lung lobe, interstitial pneumonia and suppurative bronchopneumonia were scored separately as follows: 0, absence of microscopic lesions; 1, mild interstitial pneumonia/bronchopneumonia; 2, moderate multifocal interstitial pneumonia/bronchopneumonia; 3, moderate diffuse interstitial pneumonia/bronchopneumonia; and, 4, severe interstitial pneumonia/bronchopneumonia. The sum of the score from both lesions, interstitial pneumonia and bronchopneumonia, 8 points, was the maximum possible score. Then, the average of score from the three lobes, cranial, middle and caudal, was calculated to express the result of lung evaluation. Tracheobronchial lymph node was evaluated for the presence of tingible body macrophages and lymphoid depletion. For each lesion, a maximum of 2 points was scored, being 0, absence of microscopic changes; 1, mild to moderate microscopic changes and, 2, severe microscopic changes. The final score was the sum of the score for each lesion, lymphoid depletion and presence of tingible body macrophages, with a maximum score of 4 points.

### 2.3. PCR analysis

#### 2.3.1. RNA extraction and cDNA synthesis

RNA was isolated from a total of 100 mg of lung (around 33 mg per lobule, cranial, middle and caudal) and tracheobronchial lymph node tissue samples separately homogenized with 2 ml of TRIzol™ LS Reagent using a homogenizer 150 (FisherBrand™Thermo Fisher Scientific, Barcelona, Spain), and following the manufacturer’s guidelines. Then, RNA was extracted by using the NucleoSpin® RNA virus columns kit (Macherey-Nagel, Düren, Germany) following manufacturer’s instructions. In order to remove genomic DNA, the DNase type I Ambion® TURBO-DNA-free™ kit (Life Technologies, Carlsbad, CA, USA) was used. A determination of concentration and purity of isolated RNA was conducted by Nanodrop 2000 (Thermo Fisher Scientific, Barcelona, Spain) obtaining samples with a ratio 260/280 around 2. Finally, we used 1 μl of high-quality purified RNA to generate cDNAs by Script™cDNA Synthesis Kit (BioRad, Hércules, CA, USA) following manufacturer’s specifications.

#### 2.3.2. PRRSV-1 viral load analysis in tracheobronchial lymph node and lung

Viral load for both PRRSV-1 strains, Lena and 3249, was quantified by RT-qPCR using LSI™ VetMAX™ PRRSV EU/NA 2.0 kit (Invitrogen). Amplifications were run in duplicate for each sample in the MyiQ™ 2 Two Color Real-Time PCR Detection System (BioRad) for 5 minutes (min) at 50°C, 10 min at 95°C followed by 40 cycles of 3 seconds (s) at 95°C and 30 cycles at 60°C for 30 s. An inter-run calibrator sample with a known quantification cycle (Cq) value was introduced in each plate to detect inter-run variations. To not overestimate the number of viral particles, results of PRRSV genome in lung and tracheobronchial lymph node were expressed in Cq (Kuzemtseva et al., 2014).

#### 2.3.3. Relative quantification of immune checkpoints

The comparative 2^-ΔΔCT^ method was used for the relative quantification of the porcine immune checkpoints (*CD28*, *CTLA4*, *PD1*, *PDL1*, *CD226*, *TIGIT*, *TIM3*, *LAG3*, *IDO1*, *TNFRSF9*, *SELL*, *ICOS* and *CD40*). Cq values from target genes was normalized to the Cq values from the reference genes (Pfaffl MW, 2007). The number of optimal stable reference genes requiered for normalization was determined using GeNorm analysis (qbase+2.6.1 software, Biogazelle, Zwijnaarde, Belgium^1^ Vandesompele et al., 2002). Thus, the most stable refence genes were *RPL4*, *PPIA* and *B2M* for lung and *RPL4*, *HPRT1* and *TBP* for tracheobronchial lymph node. The primers sequences for porcine reference genes and immune checkpoints are listed in Table 1. *PPIA*, *B2M*, *CD28*, *PD1*, *PDL1*, *CD226*, *TIGIT*, *TIM3*, *SELL*, *TNFRSF9*, *ICOS* and *CD40* primers were designed using the on-line *Primer3Plus* tool^2^ (Untergasser et al., 2007). The iTaq™ Universal SYBR Green Supermix kit (BioRad) was employed according to the supplier’s instructions. Reactions were performed in duplicate by using 50 ng of cDNA from each sample and 0.5 μM of each primer in the MyiQ™ 2 Two Color Real-Time PCR Detection System (BioRad) for 20 s at 95°C for polymerase activation, followed by 40 cycles for denaturalization (15 s, 95°C) and annealing/extension (30 s, 60°C). To verify the specificity of amplicons, an analysis of melting curve was performed (65–95°C). An inter-run calibrator sample with a known Cq value was included in each plate to check the quality of the retro-transcription and to detect inter-run variations. Results from relative quantification of porcine immune checkpoints from tissues of 3249- and Lena-infected animals was performed by comparison of the value from each infected animal versus the average of control animals at each specific time point. Data are represented as the fold change.

**Table 1:** Primer sequences of the porcine reference *genes* (*RPL4*, *PPIA*, *B2M*, *HPRT1*, and *TBP*) and target genes (*CD28*, *CTLA4*, *PD1*, *PDL1*, *CD226*, *TIGIT*, *TIM3*, *LAG3*, *IDO1*, *TNFRSF9*, *SELL*, *ICOS*, and *CD40*).

Genes	Type	Sequences	Reference
*RPL4*	Reference gene	F 5′-CAAGAGTAACTACAACCTTC-3′	Nygard et al. (2007)
		R 5′-GAACTCTACGATGAATCTTC-3′	
*PPIA*	Reference gene	F 5′-CGCGTCTCCTTCGAGCTGTTT-3′	Self-designed
		R 5′-GCGTGTGAAGTCACCACCCT-3´	
*B2M*	Reference gene	F 5′-ACTTTTCACACCGCTCCAGT-3´	Self-designed
		R 5′-CGGATGGAACCCAGATACAT-3´	
*HPRT1*	Reference gene	F 5′-GGACTTGAATCATGTTTGTG-3´	Nygard et al. (2007)
		R 5′-CAGATGTTTCCAAACTCAAC-3′	
*TBP*	Reference gene	F 5′-ACGTTCGGTTTAGGTTGCAG −3′	Uddin et al. (2011)
		R 5′-GCAGCACAGTACGAGCAACT-3′	
*CD28*	Target gene	F 5′-CCCCTCAATTCAAGTAACAGGAAAC-3′	Self-designed
		R 5′-ATGCCCGGAACTCCTTTGAG-3′	
*CTLA4*	Target gene	F 5′-TCTTCATCCCTGTCTTCTCCAAA-3′	Yue et al. (2014)
		R 5′-GCAGACCCATACTCACACACAAA-3′	
*PD1*	Target gene	F 5′-AGCCCAAGCACTTCATCCTC-3′	Self-designed
		R 5′-TGTGGAAGTCTCGTCCGTTG-3′	
*PDL1*	Target gene	F 5′-GTGGAAAAATGTGGCAGCCG-3′	Self-designed
		R 5′-TGCTTAGCCCTGACGAACTC-3′	
*CD226*	Target gene	F 5′-TGGAGGAGCAGCTTTGTTGTT-3′	Self-designed
		R 5′-TTTCTGTCTCCTTCTCCTTCTCTT-3′	
*TIGIT*	Target gene	F 5′-TCACGTGGGCCAGAAAGAAATC-3′	Self-designed
		R 5′-CCAATGCTGGCGGGTATTCT-3′	
*TIM3*	Target gene	F 5′-TTCGACGGGAGCAGTAAAGC-3′	Self-designed
		R 5′-AGGGCAGGACACAGTCAAAG-3′	
*LAG3*	Target gene	F 5′-CTCCTCCTGCTCCTTTTGGTT-3′	Yue et al. (2014)
		R 5′-CAGCTCCCCAGTCTTGCTCT-3′	
*IDO1*	Target gene	F 5′-GGCACTTGATTGGTGGTCTC-3′	Bacou et al. (2017)
		R 5′-GCAATCCAAGCATCGTAAGG-3′	
*SELL*	Target gene	F 5′-CCTAGTCCGATATGTCAAAAACTGG-3′	Self-designed
		R 5′-TCATCCATGCTTCTCTGAGACTT-3′	
*TNFRSF9*	Target gene	F 5′-TTGCCAGCAAGGTCAAGAGT-3′	Self-designed
		R 5′-AGCCAAAGAACAGTCCGTCC-3′	
*ICOS*	Target gene	F 5′-CCAGCGTGCATGACCCTAAT-3′	Self-designed
		R 5′-TTGCGGGTCACATCTGTTGG-3′	
*CD40*	Target gene	F 5′-GTTTGAAGACCTGGTCAAGGGTA-3′	Self-designed
		R 5′-CATGTGCCGCAATTTGAGGAT-3′	

### 2.4. Statistical analyses

Differences between viral load in lung and tracheobronchial lymph node, as well as the expression of immune checkpoints in these tissues were estimated for approximate normality of distribution by the D’Agostino & Pearson omnibus normality test, and accordingly, followed by the Mann Whitney’s U non-parametric mean comparisons test or the Student’s t-unpaired test. Correlation between the expression of the different immune checkpoints in lung and tracheobronchial lymph node from both groups were assessed by Spearman test, considering relevant those of *ρ* > 0.55 and *p* ≤ 0.05. Additionally, correlations between the expression of immune checkpoints and the transcription factor *FOXP3,* and the macroscopic and microscopic score in lung and tracheobronchial lymph node from the same animals from a parallel study (Ruedas-Torres et al., 2021a) were performed. Figures and data analyses were performed with GraphPad Prism 7.0 software (GraphPad Prism software 7.0, Inc., San Diego, CA, USA). *p* value lower than 0.05 was considered statistically significant, indicated with ‘*’ (*p* ≤ 0.05) and ‘**’ (*p* ≤ 0.01). Data are presented as the median ± interquartile range (IQR).

## 3. Results

### 3.1. Virulent PRRSV-1 Lena strain induced marked lesion in lung but not in tracheobronchial lymph node

**Figure 1:**
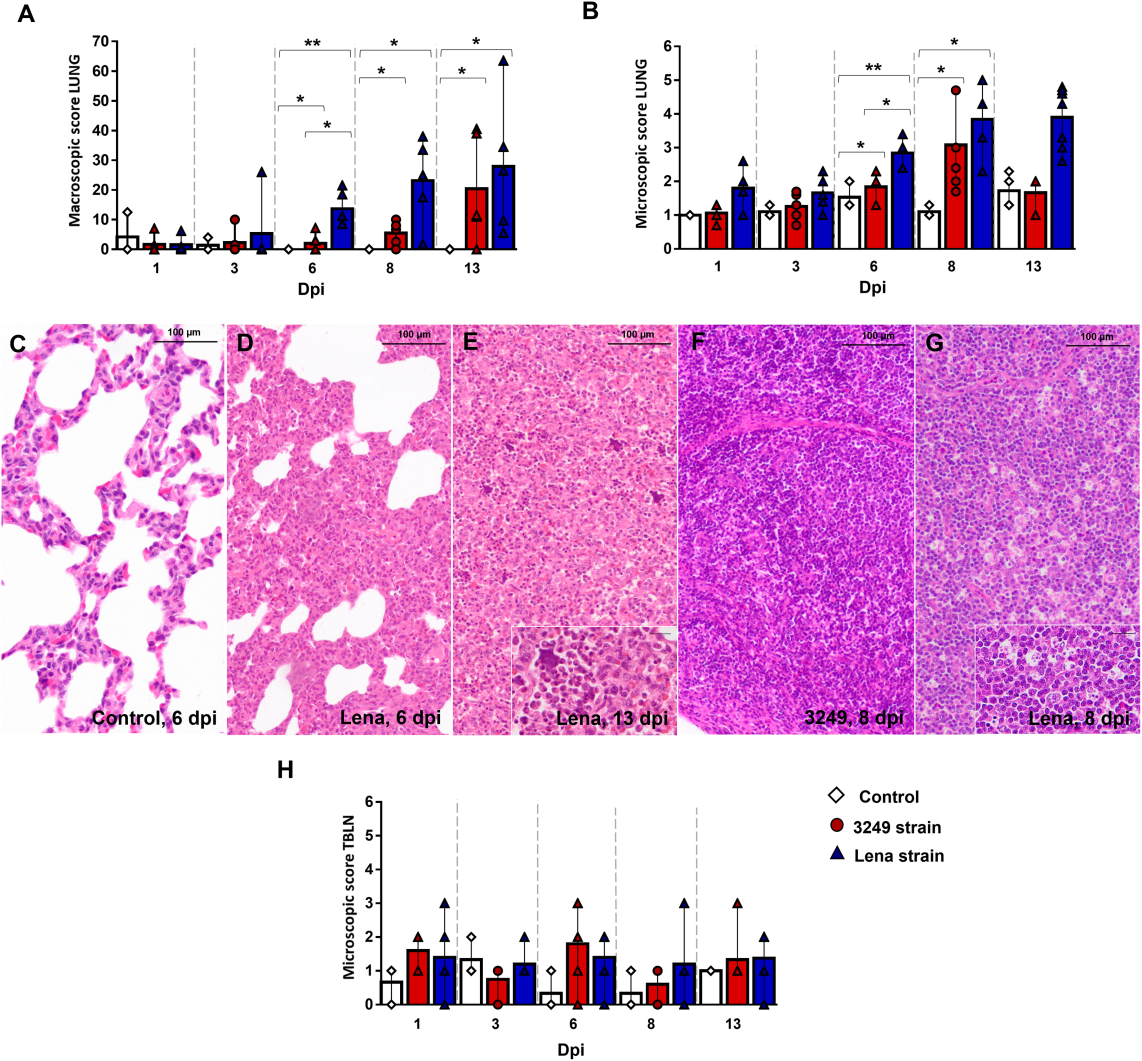
Gross and histological findings in lung and tracheobronchial lymph node. Graphs represents macroscopic **(A)** and microscopic score in lung **(B)** from control (white diamonds), 3249- (red circles) and Lena- (blue triangles) infected groups. Lung from a control animal **(C)** and from Lena-infected animals euthanized at 6 dpi **(D)** and 13 dpi **(E)**, showing severe interstitial pneumonia and bronchopneumonia, respectively. Inset shows neutrophils and cell debris filling alveolar lumen (bar 20 μm). Tracheobronchial lymph node from 3249- **(F)** and Lena- **(G)** infected animals euthanized at 8 dpi. Inset shows numerous tingible body macrophages (bar 20 μm). Graphs represents microscopic score in tracheobronchial lymph node (TBLN) **(H)**. Columns represent the median with interquartile range (IQR). Significant differences between groups in lung are represented (* *p* ≤ 0.05 and ** *p* ≤ 0.01). Dpi, days post-infection.

Gross and histological findings for lung and tracheobronchial lymph node have been previously reported (Rodríguez-Gómez et al., 2019; Ruedas-Torres et al., 2021a). Macroscopic score of lungs is represented in Figure 1A (see Rodríguez-Gómez et al., 2019 for more details). Shortly, rubbery consistency together with tan-mottled and consolidated areas were observed in the lungs from both PRRSV-1-infected groups from 6 dpi onwards. From this time-point and until the end of the experiment, lungs from Lena-infected pigs exhibited more severe lesions than 3249-infected animals (Figure 1A). Histologically, higher severity of lung lesion was observed in PRRSV-1-infected piglets compared with control group (Figures 1B–E). In particular, virulent Lena strain induced a more severe and earlier onset of lung lesion from 6 dpi onwards as consequence of severe interstitial pneumonia, characterized by thickening of the alveolar septa (Figures 1B,D), together with extensive foci of suppurative bronchopneumonia composed of neutrophils, cell debris and mucus filling the bronchial, bronchiolar and alveolar lumen (Figure 1E). By contrast, no relevant differences between experimental groups were found in the gross and microscopic examination for tracheobronchial lymph node upon PRRSV-1 infection (Figures 1F,H), nevertheless, an increase in the number of tingible body macrophages and/or severe lymphoid depletion were observed in particular 3249- and Lena-infected animals (Figure 1G; Ruedas-Torres et al., 2021a).

### 3.2. PRRSV viral load in lung and tracheobronchial lymph node was higher in Lena-infected animals

**Figure 2:**
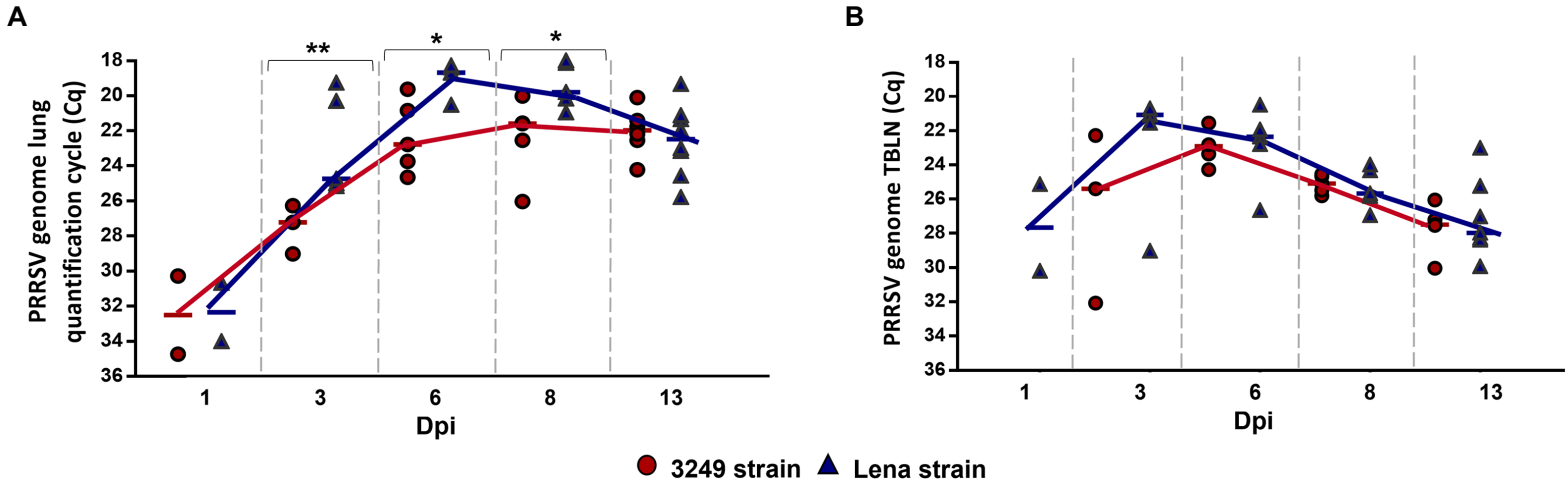
Graphs represent PRRSV viral load in lung **(A)** and tracheobronchial lymph node (TBLN) **(B)** from 3249- (red circles) and virulent Lena- (blue triangles) infected groups. PRRSV viral load is represented in quantification cycle (Cq). Significant differences between groups in lung are represented (* *p* ≤ 0.05 and ** *p* ≤ 0.01). Dpi, days post-infection.

All piglets were negative by RT-qPCR at day 0, and control group remained negative throughout the experiment. A comparable kinetics of PRRSV replication was found for both PRRSV-1 strains in the lung and tracheobronchial lymph node tissues, but viral load was higher and earlier in virulent Lena-infected piglets, particularly at lung level (Figures 2A,B). PRRSV-1 was detected in the lung of two out of five piglets from both infected groups at 1 dpi (Cq from 3249- and Lena-infected group, 34.21, IQR 4.95; and 32.34, IQR 3.34; respectively), reaching the highest level of viral replication at 6 dpi (Cq 18.68, IQR 1.27) and 8 dpi (Cq 22.07, IQR 3.89) for Lena- and 3249-infected piglets, respectively (Figure 2A). By contrast to lung, PRRSV-1 was only detected in Lena-infected piglets at 1 dpi (2 of 5 pigs) in tracheobronchial lymph node, displaying a maximum PRRSV viral load at 3 dpi with all 5 Lena-infected animals being positive (Figure 2B). In low virulent 3249-infected animals, PRRSV was first detected at 3 dpi in 3 out of 5 infected animals, presenting lower values than those determined in Lena group, and reaching the peak in viral replication at 6 dpi. PRRSV peak viral replication was followed in both infected groups by a drop until the end of the study (13 dpi; Figure 2B; Sánchez-Carvajal et al., 2020; Ruedas-Torres et al., 2021a for more details).

### 3.3. Higher expression of CTLA4 was detected in lung and tracheobronchial lymph node at the end of the study compared with CD28 in both infected groups

**Figure 3:**
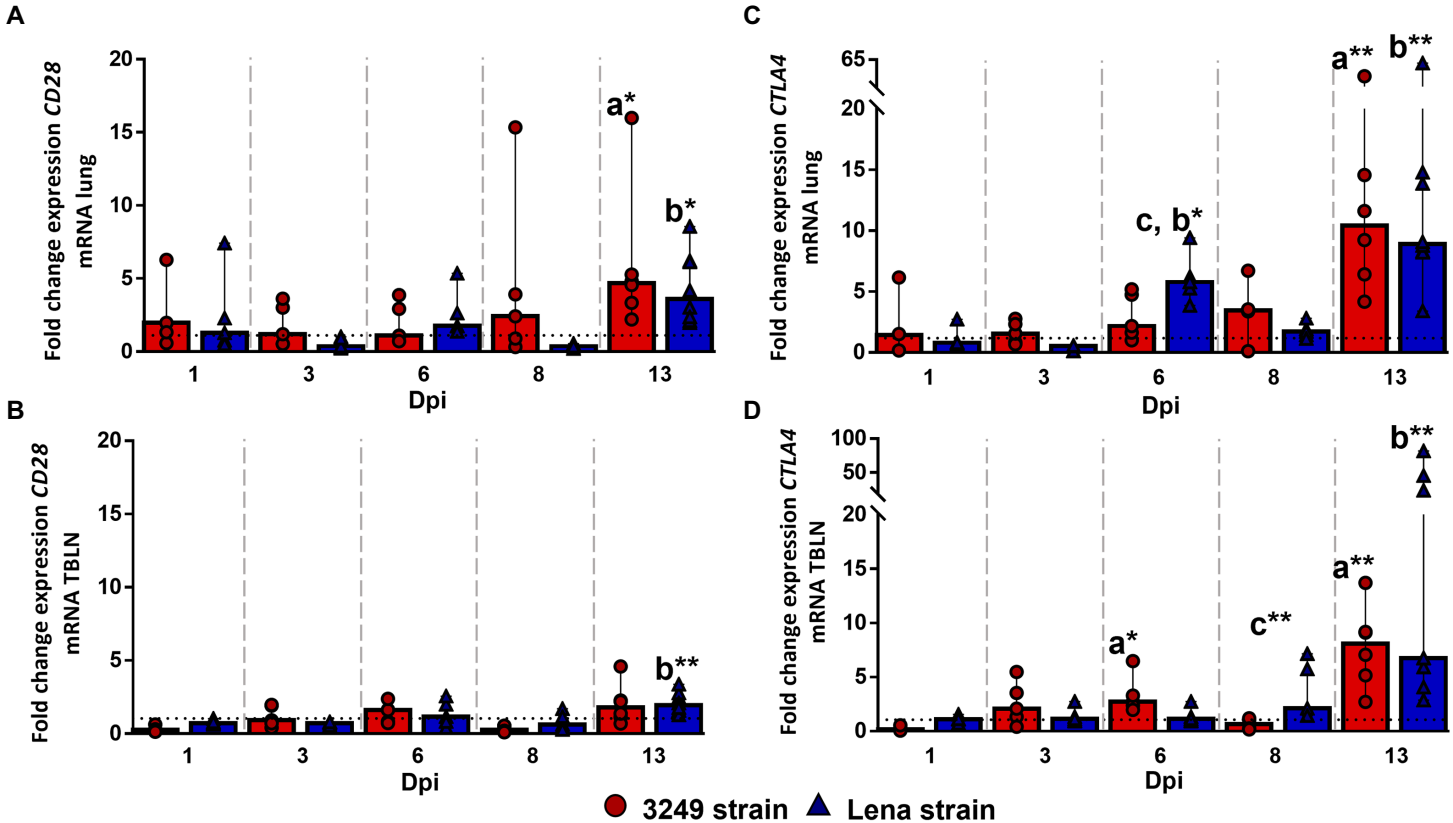
Relative mRNA expression of *CD28* and *CTLA4* mRNA. Graphs show fold change expression of *CD28* mRNA in lung **(A)** and tracheobronchial lymph node (TBLN) **(B)**, and of *CTLA4* mRNA in lung **(C)** and tracheobronchial lymph node (TBLN) **(D)**. Columns represent median with interquartile range (IQR). Individual values for each animal from low virulent 3249- (red circles) and virulent Lena- (blue triangles) infected animals are represented. Dashed line shows the expression of *CD28* and *CTLA* mRNA from control group in each organ. “a” indicates a significant difference between the 3249 and control groups, “b” between Lena and control groups and “c” between Lena and 3249 groups (* *p* ≤ 0.05 and ** *p* ≤ 0.01). Dpi, days post-infection.

Kinetics of *CD28* gene expression did not show changes in the lung and tracheobronchial lymph node from both PRRSV-1-infected groups compared with control group during the first week post-infection. In the case of lung tissue, a slight increase was found for Lena and 3249 groups, with significant differences when compared with control group, during the second week post-infection (13 dpi; *p* ≤ 0.05; fold change at 13 dpi from Lena-infected group 3.60, IQR 4.03; and from 3249-infected group 4.69, IQR 4.90; Figure 3A). Likewise, significant differences were observed in the tracheobronchial lymph node from Lena-infected animals in comparison with control animals at 13 dpi (fold change 1.93, IQR 1.12; *p* ≤ 0.01; Figure 3B).

*CTLA4* gene expression followed a similar kinetics in the lung and tracheobronchial lymph node from both PRRSV-1-infected groups. Regarding to the lung, an up-regulation of *CTLA4* gene was observed at 6 dpi (fold change 5.78, IQR 3.25) in virulent Lena-infected piglets (*p* < 0.05 with respect to 3249 and control groups), followed by an increased in both infected groups at 13 dpi (fold change 8.91, IQR 6.30; *p* ≤ 0.01; and fold change 10.42, IQR 16.83; *p* ≤ 0.05; for Lena- and 3249-infected groups, respectively; Figure 3C). In the case of tracheobronchial lymph node, an increase in the expression of *CTLA4* gene was observed at 13 dpi in both PRRSV-1-infected groups, Lena (fold change 6.73, IQR 35.47; *p* ≤ 0.01) and 3249 (fold change 8.07, IQR 5.76; *p* ≤ 0.01; Figure 3D).

### 3.4. Up-regulation of TIGIT gene was observed in target organs from virulent Lena-infected animals

**Figure 4:**
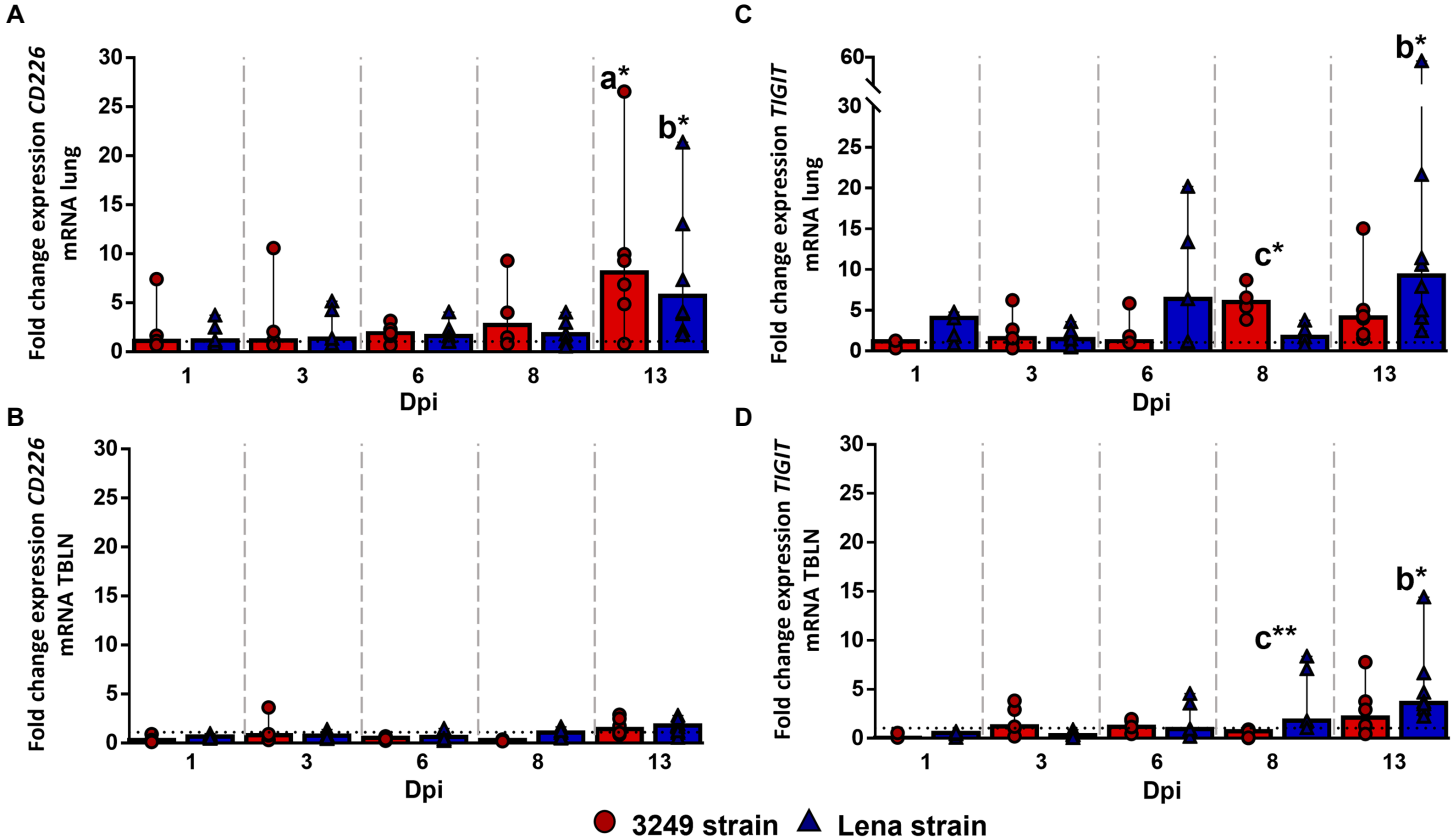
Relative mRNA expression of *CD226* and *TIGIT* mRNA. Graphs show fold change expression of *CD226* mRNA in lung **(A)** and tracheobronchial lymph node (TBLN) **(B)**, and of *TIGIT* mRNA in lung **(C)** and tracheobronchial lymph node (TBLN) **(D)**. Columns represent median with interquartile range (IQR). Individual values for each animal from low virulent 3249- (red circles) and virulent Lena- (blue triangles) infected animals are presented. Dashed line represents the expression of *CD226* and *TIGIT* mRNA from control group in each organ. “a” indicates a significant difference between the 3249 and control groups, “b” between Lena and control groups and “c” between Lena and 3249 groups (* *p* ≤ 0.05 and ** *p* ≤ 0.01). Dpi, days post-infection.

*CD226* gene expression remained at baseline or below control group in the lung, with a remarkable increase in Lena- and 3249-infected groups at 13 dpi (fold change for Lena- and 3249-infected groups, 5.70, IQR 10.40; and 8.08, IQR 10.25; respectively), finding significant differences between PRRSV-1-infected and control piglets (*p* ≤ 0.01; Figure 4A). *CD226* gene expression remained at low levels in the tracheobronchial lymph node from both Lena- and 3249-infected piglets increasing slightly at 13 dpi with a wide individual variability (fold change for Lena- and 3249-infected groups 1.77, IQR 1.26; and 1.42, IQR 1.59; respectively; Figure 4B).

A significant up-regulation of *TIGIT* (T cell immunoreceptor with Ig and ITIM domains) gene expression was observed in the lung from low virulent 3249-infected animals at 8 dpi (fold change 5.99, IQR 3.92; *p* ≤ 0.05; Figure 4C), compared with Lena and control groups, followed by a mild decrease at 13 dpi (fold change 4.13, IQR 5.61; Figure 3C). In virulent Lena-infected piglets, an increase of *TIGIT* expression was observed at 6 dpi with a wide individual variability (fold change 6.38, IQR 15.64) followed by a more consistent increase at 13 dpi (fold change 9.25, IQR 14.75; *p* ≤ 0.05 with respect to control group; Figure 4C). Regarding the tracheobronchial lymph node, *TIGIT* gene expression remained at low levels in 3249-infected piglets with a slight increase at 13 dpi (fold change 2.13, IQR 3.73; Figure 4D). By contrast, a gradual increase of *TIGIT* gene expression was observed in Lena-infected piglets from 8 (fold change 1.80, IQR 5.92) to 13 dpi (fold change 3.62, IQR 3.72; Figure 4D). Significant differences were found between Lena- and 3249-infected groups at 8 dpi (*p* ≤ 0.01), and between Lena-infected group and control group at 13 dpi (*p* ≤ 0.05; Figure 4D).

### 3.5. Strong up-regulation of PDL1 was observed in the lung from virulent Lena-infected group at 2 week post-infection

**Figure 5:**
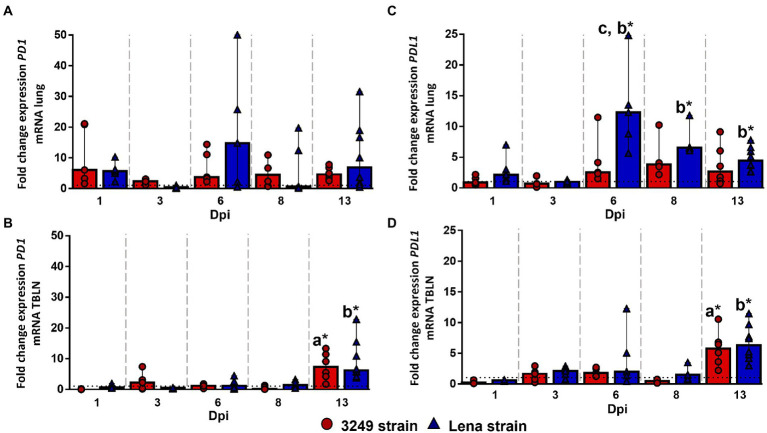
Relative mRNA expression of *PD1* and *PDL1* mRNA. Graphs show fold change expression of *PD1* mRNA in lung **(A)** and tracheobronchial lymph node (TBLN) **(B)**, and of *PDL1* mRNA in lung **(C)** and tracheobronchial lymph node (TBLN) **(D)**. Columns represent median with interquartile range (IQR). Individual values for each animal from low virulent 3249- (red circles) and virulent Lena- (blue triangles) infected animals are presented. Dashed line represents the expression of *PD1* and *PDL1* mRNA from control group in each organ. “a” indicates a significant difference between the 3249 and control groups, “b” between Lena and control groups and “c” between Lena and 3249 groups (* *p* ≤ 0.05 and ** *p* ≤ 0.01). Dpi, days post-infection.

No statistically significant differences were observed for *PD1* gene expression in the lung along the study (Figure 5A). However, *PD1* expression was found to be higher in low virulent 3249-infected group than in control group from 1 dpi to the end of the study, maintaining the level of expression over the time (fold change around 5.00–6.00; Figure 5A). In the case of virulent Lena-infected piglets, the expression of *PD1* was enhanced at 1, 6 and 13 dpi reaching at 6 dpi a fold increase of 14.78 (IQR 36.52) but showing a marked individual variability (Figure 5A). In the case of the tracheobronchial lymph node, both PRRSV-1-infected groups followed a similar trend regarding to *PD1* gene expression (Figure 5B), presenting a significant up-regulation at 13 dpi (fold change for Lena- and 3249-infected groups, 6.22, IQR 9.72; and 7.29, IQR 8.29; respectively), which was statistically significant with respect to control group (*p* ≤ 0.05; Figure 5B).

The kinetics of *PDL1* gene expression was completely different for 3249- and Lena-infected piglets. Whereas *PDL1* gene expression remained nearly constant along the study, with a slight to moderate increase at 6, 8 and 13 dpi in 3249 group (fold change 2.53, IQR 5.89; 3.78, IQR 6.18; and 2.64, IQR 5.78; respectively; Figure 5C), *PDL1* expression underwent a strong up-regulation at 6 dpi in virulent Lena-infected piglets (fold change 12.29, IQR 11.93), followed by a progressive drop until 13 dpi (fold change at 8 dpi and 13 dpi, 6.55, IQR 2.96; and 4.42, IQR 3.52; respectively; Figure 5C). Significant differences were observed between Lena-infected group and control group at 6, 8, and 13 dpi and between virulent Lena- and low virulent 3249-infected groups at 6 dpi (*p* ≤ 0.05; Figure 5C). In the case of tracheobronchial lymph node, *PDL1* gene expression was similar in both PRRSV-1-infected groups (Figure 5D), remaining at baseline or below control group with a significant increase at 13 dpi (fold change for Lena- and 3249-infected groups, 6.33, IQR 4.86; and 5.76, IQR 4.47; respectively), and showing significant differences in comparison with control group (*p* ≤ 0.05; Figure 5D).

### 3.6. Coinhibitory molecules TIM3, LAG3, and IDO1 were mainly expressed in target organs from virulent Lena-infected animals

**Figure 6:**
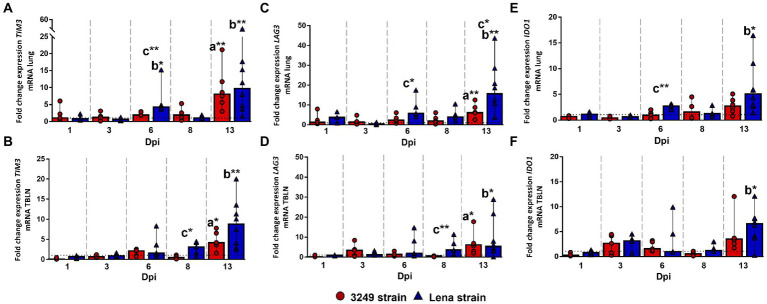
Relative mRNA expression of *TIM3*, *LAG3* and *IDO1* mRNA. Graphs show fold change expression of *TIM3* mRNA in lung **(A)** and tracheobronchial lymph node (TBLN) **(B)**, of *LAG3* mRNA in lung **(C)** and tracheobronchial lymph node (TBLN) **(D)**, and of *IDO1* in lung **(E)** and tracheobronchial lymph node (TBLN) **(F)**. Columns represent median with interquartile range (IQR). Individual values for each animal from low virulent 3249- (red circles) and virulent Lena- (blue triangles) infected animals are showed. Dashed line represents the expression of *TIM3*, *LAG3* and *IDO1* mRNA from control group in each organ. “a” indicates a significant difference between the 3249 and control groups, “b” between Lena and control groups and “c” between Lena and 3249 groups (* *p* ≤ 0.05 and ** *p* ≤ 0.01). Dpi, days post-infection.

TIM3 molecule (T cell immunoglobulin and mucin-domain containing-3, encoded by *HAVCR2* gene, also known as *TIM3*), *LAG3* (lymphocyte-activation gene 3) and *IDO1* (indoleamine 2,3-dioxygenase 1) gene expression followed a similar kinetics in both studied tissues, lung and tracheobronchial lymph node, although the fold increase for *TIM3* and *LAG3* was higher in the lung (Figure 6). In this organ, a moderate increase was observed in the expression of *TIM3*, *LAG3* and *IDO1* at 6 dpi in virulent Lena-infected piglets (fold change 4.27, IQR 6.31; 5.75, IQR 8.46; and 2.72, IQR 0.87; respectively), showing significant differences with respect to low virulent 3249-infected group and/or control group (Figures 6A,C,E). After a mild to moderate decrease observed at 8 dpi, a further rise was observed at 13 dpi (fold change 9.70, IQR 13.13; 15.63, IQR 15.87; and 5.06, IQR 6.93; for *TIM3*, *LAG3* and *IDO1*, respectively), presenting significant differences with respect to the other experimental groups (Figures 6A,C,E). The expression of these coinhibitory molecules in the lung from low virulent 3249-infected animals was similar to control group remaining low and steady but significantly increasing at the end of the study (fold change 8.03, IQR 9.14; 6.08, IQR 7.04; and 2.72, IQR 2.62; for *TIM3*, *LAG3* and *IDO1*, respectively; Figures 6A,C,E).

In tracheobronchial lymph node, a progressive increase was observed in virulent Lena-infected animals from 6 dpi onwards, displaying significant differences at 8 dpi (fold change 3.11, IQR 3.20; and 3.58, IQR 6.52; for *TIM3* and *LAG3*, respectively) and 13 dpi (fold change 8.03, IQR 9.14; 5.30, IQR 14.99; and 6.57, IQR 3.88; for *TIM3*, *LAG3* and *IDO1*, respectively; Figures 6B,D,F). In low virulent 3249-infected animals a mild to moderate enhancement in the expression of some of these coinhibitory molecules was observed at 3 dpi, showing a significant increase at the end of the study (13 dpi; fold change 6.12, IQR 6.89; 6.12, IQR 6.36; and 3.49, IQR 3.43; for *TIM3*, *LAG3* and *IDO1*, respectively), which were significant when compared to control group (*p* ≤ 0.05; Figures 6B,D,F).

### 3.7. Costimulatory molecules TNFRSF9, SELL, ICOS, and CD40 were mainly activated in lung but not in tracheobronchial lymph node from PRRSV-1-infected animals

**Figure 7:**
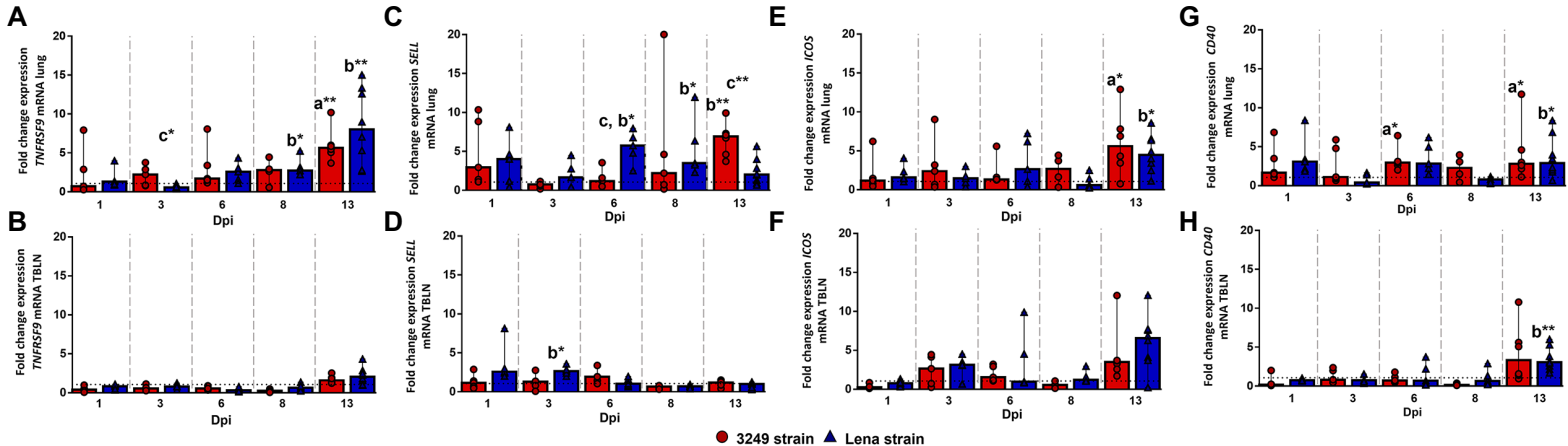
Relative mRNA expression of *TNFRSF9*, *SELL, ICOS* and *CD40* mRNA. Graphs show fold change expression of *TNFRSF9* mRNA in lung **(A)** and tracheobronchial lymph node (TBLN) **(B)**, of *SELL* mRNA in lung **(C)** and tracheobronchial lymph node (TBLN) **(D)**, of *ICOS mRNA* in lung **(E)** and tracheobronchial lymph node (TBLN) **(F)** and of *CD40* mRNA in lung **(G)** and tracheobronchial lymph node (TBLN) **(H)**. Columns represent median with interquartile range (IQR). Individual values for each animal from low virulent 3249- (red circles) and virulent Lena- (blue triangles) infected animals are depicted. Dashed line represents the expression of *TNFRSF9*, *SELL, ICOS* and *CD40* mRNA from control group in each organ. “a” indicates a significant difference between the 3249 and control groups, “b” between Lena and control groups and “c” between Lena and 3249 groups (* *p* ≤ 0.05 and ** *p* ≤ 0.01). Dpi, days post-infection.

The expression of *TNFRSF9* (TNF receptor superfamily member 9) gene in the lung followed a similar kinetics for both PRRSV-1 strains, 3249 and Lena, increasing progressively from 6 to 13 dpi when the highest level of expression was found (fold change 8.02, IQR 9.63; and 5.65, IQR 2.31; for Lena- and 3249-infected groups, respectively; Figure 7A). Significant differences were found at 13 dpi between both infected groups with respect to control group (*p* ≤ 0.01). The expression of *TNFRSF9* in the tracheobronchial lymph node from both infected groups remained plateau throughout the study, displaying a mild, no significant, increase at 13 dpi (Figure 7B).

*SELL* (L-sectin or CD62L) gene expression was up-regulated in the lung from low virulent 3249-infected piglets as early as 1 dpi, but no significant differences were found due to a wide individual variability (fold change 2.95, IQR 8.34; Figure 7C). After that, *SELL* gene expression returned to basal level, increasing progressively until the end of the study (13 dpi; fold change 6.91, IQR 3.57), when significant differences were observed between 3249-infected group with respect to Lena-infected group and control group (*p* ≤ 0.01; Figure 7C). In the case of virulent Lena-infected group, the expression of *SELL* followed a two-peak curve with the first peak being observed at 1 dpi (fold change 4.02, IQR 5.56), but with wide individual variability, and the second peak at 6 dpi (fold change 5.57, IQR 3.69; *p* ≤ 0.05), decreasing onwards (Figure 7C). In tracheobronchial lymph node, *SELL* gene expression remained at baseline or below control group with a slight increase at 1 and 3 dpi for Lena group (fold change at 3 dpi, 2.66, IQR 1.01; *p* ≤ 0.05; Figure 7D).

In the lung, *ICOS* gene expression showed a mild progressive increase until the end of the study (13 dpi; fold change 5.06, IQR 6.24), when significant differences were found with respect to control group (*p* ≤ 0.05; Figure 7E). A similar dynamic was found for Lena-infected piglets, which presented a moderate drop in the expression of *ICOS* at 8 dpi followed by a significant up-regulation at 13 dpi (fold change 4.45, IQR 3.77; *p* ≤ 0.05; Figure 7E). The kinetics of *ICOS* expression was similar for both PRRSV-1-infected groups, showing a two peaks curve in the tracheobronchial lymph node (Figure 7F). The first peak was detected at 3 dpi (fold change 3.14, IQR 2.13; and 2.64, IQR 3.79; for Lena- and 3249-infected groups, respectively) with the second one being observed at 13 dpi, more importantly in Lena-infected animals (fold change 6.57, IQR 3.88; and 3.49, IQR 3.43; for Lena- and 3249-infected group, respectively; Figure 7F). However, due to the wide individual variability no significant differences were detected during the whole study (Figure 7F).

Regarding *CD40* gene expression a mild to moderate increase was irregularly observed along the study, showing statistically significant differences with respect to control group at 6 and 13 dpi in the lung of low virulent 3249-infected piglets (fold change at 6 and 13 dpi, 2.96, IQR 2.45; and 2.08, IQR 4.48, respectively; *p* ≤ 0.05) and at 13 dpi in the lung of virulent Lena-infected piglets (fold change 2.89, IQR 4.47; *p* ≤ 0.05; Figure 7G). The expression of *CD40* remained at baseline or below control group in the tracheobronchial lymph node from both infected groups, displaying an increase at the end of the study (fold change, 3.32, IQR 5.61; and 3.05, IQR 2.66; for 3249- and Lena-infected group, respectively; *p* ≤ 0.01 between Lena and control group; Figure 7H).

### 3.8. Immune checkpoints were highly correlated among them and with the expression of FOXP3 in both infected groups along the study

**Figure 8:**
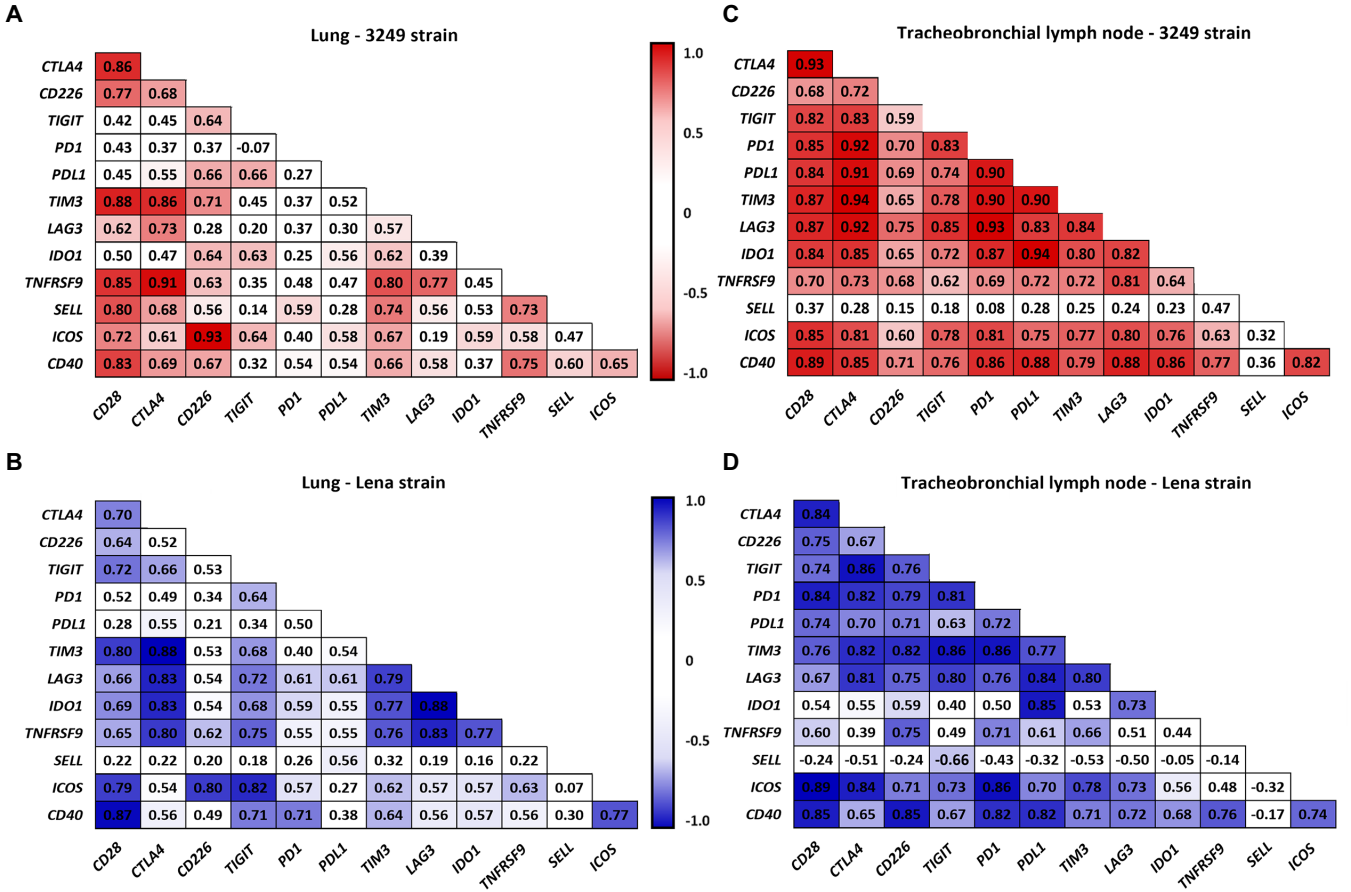
Correlation between the expression of immune checkpoints in lung for 3249- **(A)** and Lena- **(B)** infected groups and in tracheobronchial lymph node for 3249- **(C)** and Lena- **(D)** infected groups are shown. Significant correlations for 3249 and Lena strains are represented with red and blue color, respectively (ρ > 0.55, *p* ≤ 0.05).

**Figure 9:**
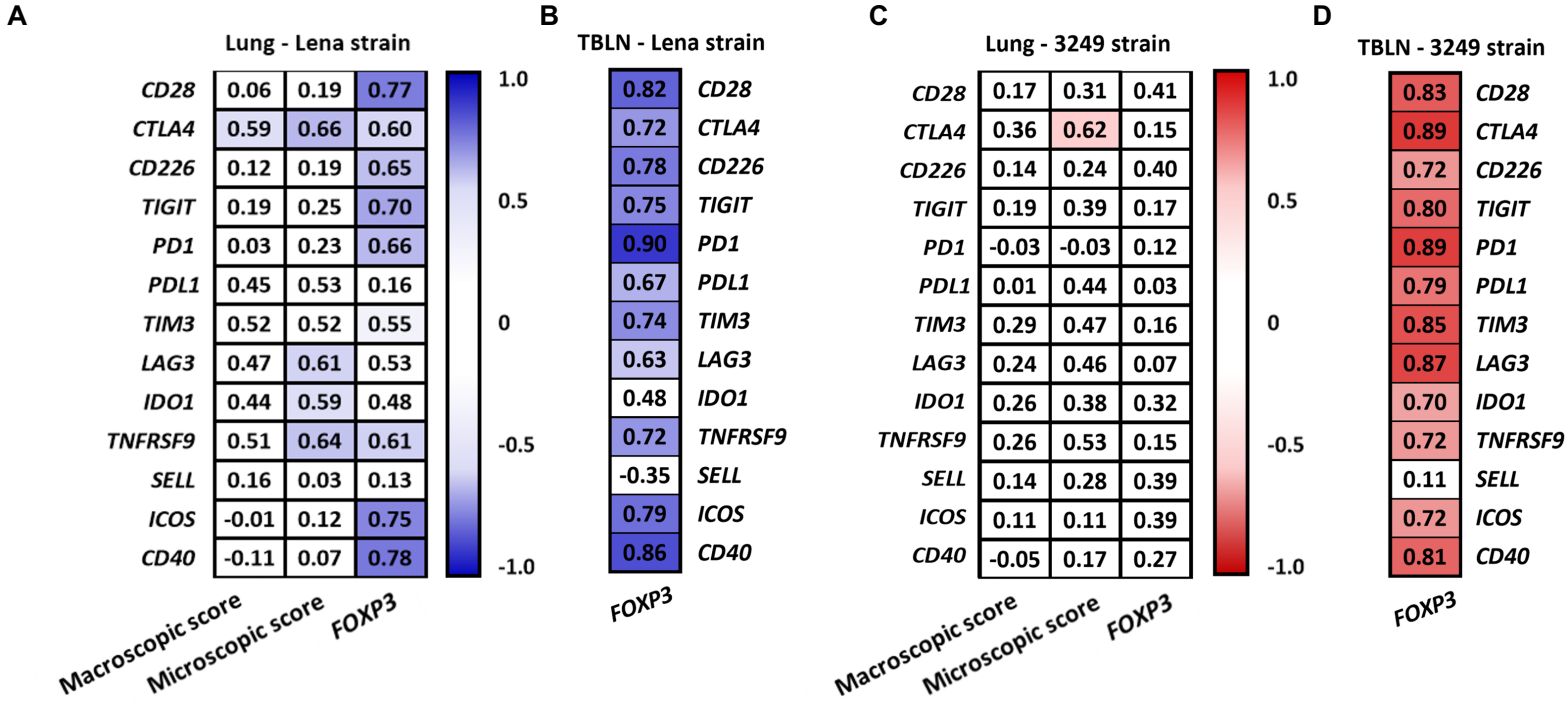
Correlation found between the expression of immune checkpoints, the macroscopic and the microscopic score and the expression of *FOXP3* in lung from Lena- **(A)** and 3249-infected group **(C).** Correlation found between the expression of immune checkpoints and the expression of *FOXP3* in the tracheobronchial lymph node (TBLN) from Lena- **(B)** and 3249-infected group **(D).** Significant correlations for Lena and 3249 strains are represented with blue and red color, respectively (ρ > 0.55, *p* ≤ 0.05).

Figure 8 summarizes the correlations between the expression of the different analyzed immune checkpoints in the lung (Figures 8A,B) and tracheobronchial lymph node (Figures 8C,D) from low virulent 3249 and virulent Lena strain. Strong correlations between the expression of different immune checkpoints were found in both organs. Additionally, in the lung from low virulent 3249-infected animals, correlation between the expression of *CTLA4* and the microscopic lung score was found (*ρ* = 0.62 and *p* ≤ 0.05). For virulent Lena-infected animals, *CTLA4* showed correlation with macroscopic score (*ρ* = 0.66, *p* ≤ 0.05) and several immune checkpoints including *CTLA4*, *LAG3*, *IDO1* and *TNFRSF9* also showed correlation with microscopic lung lesion (*CTLA* 4 ρ = 0.66, *LAG3* 4 *ρ* = 0.61, *IDO1 ρ* = 0.59 and *TNFRSF9 ρ* = 0.64, *p* ≤ 0.05; Figure 9A). In the case of 3249-strain only *CTLA4* immune checkpoint showed correlation with the microscopic lung lesion (ρ = 0.62, *p* ≤ 0.05; Figure 9C). Moreover, high correlation between various immune checkpoints and the expression of the transcription factor *FOXP3* was found in the lung from virulent Lena-infected animals (Figure 9A). The expression of the transcription factor *FOXP3* also showed high correlation with immune checkpoints in the tracheobronchial lymph node from Lena- and 3249-infected groups (Figures 9B,D, respectively).

## 4. Discussion

One of the intriguing features of PRRSV infection is its ability to modulate the host immune response to its favor (Butler et al., 2014; Lunney et al., 2016). In this sense, many processes have been postulated as strategies carried out by PRRSV to ensure its distribution and replication in target organs, such as regulated cell death induction, suppression of interferon (IFN), modulation of cytokine expression and impairment of antigen presentation (Huang et al., 2015; Ruedas-Torres et al., 2020; Sánchez-Carvajal et al., 2021b). Antigen presentation constitutes a mandatory immune process to activate naïve T cells, initiating the adaptive immune response. During this process, costimulatory and coinhibitory molecules participate regulating the activation and termination of T-cell response (Guermonprez et al., 2002; Jiang and Chess, 2006; Viganò et al., 2012; Attanasio and Wherry, 2016; Wykes and Lewin, 2018; Cai et al., 2020). Thus, the lack of costimulatory signals or exuberant coinhibitory signals lead to anergy or tolerance (Sckisel et al., 2015). Many coinhibitory molecules such as CTLA4, PD1/PDL1, LAG3, CD200:CD200R have been evaluated during acute viral infection in pigs, suggesting an effect of these molecules to prevent overstimulation of T cell and/or immunopathology in peripheral organs (Yue et al., 2014; Richmond et al., 2015a,b; Yue et al., 2015; Cai et al., 2020; Ruedas-Torres et al., 2021b). However, to the authors’ knowledge, few studies have considered the expression of costimulatory and coinhibitory molecules in target organs upon PRRSV infection (Yue et al., 2014; Richmond et al., 2015a,b; Yue et al., 2015; Cai et al., 2020; Ruedas-Torres et al., 2021b). Thus, the present work evaluates the relative expression of costimulatory (*CD28*, *CD226*, *TNFRSF9*, *SELL*, *ICOS* and *CD40*) and coinhibitory (*CTLA4*, *TIGIT*, *PD1/PDL1* axis, *TIM3*, *LAG3*, and *IDO1*) molecules in lung and tracheobronchial lymph node upon an experimental infection with two PRRSV-1 strains of different virulence, virulent Lena and low virulent 3249 strains, during the first 2 weeks post-infection.

One of the essential costimulatory signals involved in T-cell activation is the interaction between CD80/CD86 molecules on the surface of APCs and their ligand CD28 on T cells (Viganò et al., 2012). By contrast, CTLA4 is another ligand for CD80/CD86 which presents even a higher avidity than CD28, acting as a critical inhibitor of T-cell activation and proliferation in several viral infections (Collins et al., 2002; Cai et al., 2020). In our study, a minor rise in the expression of *CD28* gen was observed at the end of the study (13 dpi) in both PRRSV-1-infected groups, in contrast with a striking peak of greater magnitude in the expression of *CTLA4* in lung and tracheobronchial lymph node from both infected groups. These results likely reflect an imbalance between these costimulatory and coinhibitory molecules, that modulates the host pro-inflammatory and adaptive immune response. Furthermore, a moderate increase in *CTLA4* expression was also observed at first week post-infection (6 dpi) in both infected groups, which coincided with the peak of viral load in lung (virulent Lena strain) or tracheobronchial lymph node (low virulent 3249 strain) and correlated with the severity of lung lesion in both infected groups (*ρ* > 0.6). In this sense, CTLA4 has been reported to induce initiation of apoptosis *via* caspase-8 and caspase-3 (Contardi et al., 2005; Boise et al., 2010). Interestingly, a high rate of caspase-8 has been observed in the lung of these animals from the first week post-infection onwards in a parallel study performed by our research group (Sánchez-Carvajal et al., 2021b). All together our results evidence an increased expression of *CTLA4* gen in target organs from PRRSV-1-infected animals during the first 2 weeks post-infection associated with the inflammatory tissue-damage observed in the lung, particularly in Lena-infected piglets.

The costimulatory molecule CD226 is highly expressed on T cells and natural killer (NK) cells and binds to CD155 and CD112, expressed on APCs, among a wide range of tissue cells (Bottino et al., 2003; Dardalhon et al., 2006). This linkage enhances the cytotoxicity of T cells and NK cells (Arruga et al., 2019). However, TIGIT, the coinhibitory counterpart, may limit T-cell driven inflammation by directly competing for binding CD155 and/or CD112 molecules with higher affinity than CD226 (Anderson et al., 2016; Arruga et al., 2019). In our study, a high expression of *CD226* and *TIGIT* genes was observed at the end of the experiment in the lung from both infected groups, with *TIGIT* being also up-regulated in the tracheobronchial lymph node at that date impacting, probably, on T-cell activation (Joller et al., 2014). TIGIT expression has been associated with selective suppression of proinflammatory Th1 and Th17 cells by regulatory T cells (Tregs; Joller et al., 2014), limiting inflammatory tissue-damage in peripheral organs independently of viral clearance (Schorer et al., 2020); CD112/CD226 axis seems to play a role activating NK-cell response and contributing to viral clearance by killing infected cells during acute virus influenza infections (Redlberger-Fritz et al., 2019; Schorer et al., 2020). Therefore, these results suggest that during acute PRRSV-1 infection *CD226* and *TIGIT* might work together contributing to viral clearance and controlling an impaired proinflammatory response, mainly in virulent Lena-infected piglets, in which lung tissue was more severely affected.

Previous studies have already pointed to an up-regulation of *PD1/PDL1* axis in PRRSV-1 and PRRSV-2 infections (Richmond et al., 2015a,b; Chaudhari et al., 2020, 2021; Ruedas-Torres et al., 2021b), with decreased levels of apoptosis and anergy in porcine lymphocytes deficient in PD1 (Richmond et al., 2015b). In our study, virulent Lena-infected piglets showed a remarkable up-regulation of *PDL1* from 6 dpi onwards at lung level in association with a more severe inflammatory response. Virulent PRRSV-1 strains are characterized by inducing a strong inflammatory response linked to an increase of IFN-α/IFN-γ and other proinflammatory cytokines such as IL-1α, IL-1β and TNF-α (Weesendorp et al., 2013; Amarilla et al., 2015; Renson et al., 2017; Sánchez-Carvajal et al., 2020). Interestingly, type I IFN (IFN-α/β) and other inflammatory cytokines are able to increase the expression of PDL1 (Garcia-Diaz et al., 2017; Schönrich and Raftery, 2019). These findings indicate that *PDL1* up-regulation, although more marked in virulent Lena- than in low virulent 3249-infected piglets, could represent a physiological part of the porcine innate response induced by IFNs and/or pattern recognition receptors (PRRs) signaling-pathways (Schönrich and Raftery, 2019); however, its role during early and late stages of PRRSV infection needs further dissection. These results are in line with those from Auray and co-authors who reported an upregulation of PDL1 in DCs after treatment with different TLR ligands (Auray et al., 2020). Furthermore, the kinetics of *PDL1* gene expression in the lung of virulent Lena-infected animals was parallel to the one previously reported in the thymus from the same animals (Ruedas-Torres et al., 2020). Similar results have been previously observed in DCs from pigs infected with low and highly virulent classical swine fever virus (CSFV) strains after transcriptomic analysis, associated with high levels of death receptors as well. Up-regulation of these molecules was higher in the virulent CSFV strain and would contribute to the lymphopenia observed after CSFV infection (Auray et al., 2020). Besides playing a potential role in apoptosis phenomena, PDL1 has been hypothesized to interact with CD80 molecule competing or inhibiting its interaction with CD28 and, thus, leading to an inhibitory milieu which affects T-cells activation (Yue et al., 2015; Ruedas-Torres et al., 2021b). In the tracheobronchial lymph node from both infected groups an increase in the expression of *PD/PDL1* axis was observed at the end of the study, which could lead to a disorder on T-cell activation.

The coinhibitory molecules TIM3, LAG3 and IDO1 have been poorly examined along porcine viral diseases. An up-regulation of TIM3 and IDO1 has been described in the context of PRRSV- and CSFV-infected pigs, respectively (Hulst et al., 2013; Chaudhari et al., 2020), whereas LAG3 was reported to play a limited role during the pathogenesis of postweaning multisystemic wasting syndrome (Yue et al., 2015). In our study, *TIM3*, *LAG3* and *IDO1* were found overexpressed earlier and more markedly in the lung and tracheobronchial lymph node from virulent Lena- than low virulent 3249-infected piglets, with an enhancement in both groups at the end of the study (13 dpi). TIM3 and LAG3 have been found briefly up-regulated in activated CD4^+^ and CD8^+^ T-cells, in exhausted CD8 T-cells, Tregs, type 1 regulatory T cells and NK cells, but also in DCs in the case of TIM3 (Anderson et al., 2016). IDO1 is an immune regulatory enzyme, induced at sites of inflammation, capable of modulating the immune cell activation (Yeung et al., 2015), and preventing the viral spread in some viral infections (Schmidt and Schultze, 2014; Yeung et al., 2015). The similar kinetics showed by all three coinhibitory molecules in the present study points out to their co-expression in PRRSV-infected tissues, which together with the co-expression of other molecules such as *PDL1*, *CTLA4* or *TIGIT*, has been highlighted as a mechanism involved in enhancing their inhibitory properties (Kahan et al., 2015; Cai et al., 2020; Wolf et al., 2020). In fact, high correlation between the expression of these molecules has been found in target organs from both infected groups which reinforces our hypothesis. In this sense, although several PRRSV strains have been reported to elicit a transient increase in the frequency of CD8^+^ T cells (Gómez-Laguna et al., 2009; Morgan et al., 2013; Weesendorp et al., 2013), these cells have been reported not to be functional (Costers et al., 2009). The pathways involved in the lack of cytotoxic activity of these cells in PRRS are poorly understood, but the co-expression of the coinhibitory molecules observed in our study highlight a mechanism which might be potentially involved in this process. In addition, these molecules may be also participating in the modulation of the inflammatory response since some cytokines, such as IFN-γ and TNF-α, have been described as proinflammatory mediators inducing IDO1 expression in macrophages and DCs (Robinson et al., 2005). Indeed, the progressive rise in the expression of *TIM3*, *LAG3* and *IDO1* in the lung from virulent Lena-infected animals correlated with the higher severity of lesions observed in these animals in our study, suggesting an attempt to minimize bystander tissue damage and hindering the inflammatory response as has been proposed for other molecules along virulent PRRSV infection (Sánchez-Carvajal et al., 2021a).

*TNFRSF9*, *SELL*, *ICOS* and *CD40* costimulatory molecules showed low expression in the tracheobronchial lymph node from both PRRSV-1-infected groups which would be associated with a mild T-cell activation. However, an up-regulation of these molecules was observed from the first week post-infection onwards in the lung. TNFRSF9 (also known as 4-1BB or CD137) plays a role in the survival of diverse cell types through the interaction with its ligand TNFSF9 on DCs (4-1BBL/CD137L; Takahashi et al., 2005; Wang et al., 2009). Thus, the TNFSF9/TNFRSF9 signaling pathway plays a bidirectional role during inflammation stimulating CD4^+^ T cells to produce IFN-γ and TNF-α (Kwon, 2009; Kwon, 2012), contributing to the recruitment and activation of neutrophils (Kwon, 2009; Kwon, 2012), but also supressing inflammation by controlling regulatory activities of Tregs and DCs (Kwon, 2012). SELL is expressed on most of leukocytes and is responsible of the trafficking of leukocytes and neutrophils to site of inflammation (Raffler et al., 2005), playing a pivotal role during the clearance of acute inflammation, but under several situations, an exuberant leukocytes infiltration contributes to the severity of the tissue damage (Raffler et al., 2005). Therefore, the higher expression of *TNFRSF9* and *SELL* found in the lung from PRRSV-1-infected piglets could be related to the clearance of infected cells, cellular debris, and resolution of inflammation as an attempt to restore the normal lung homeostasis during the acute phase of PRRSV-1 infection.

ICOS belongs to the CD28 and CTLA4 receptor family mediating T-cell activation, proliferation and differentiation, being critical for follicular helper T (Tfh) cells generation and germinal center formation (Wikenheiser and Stumhofer, 2017; Qin et al., 2018). In turn, CD40 expressed on APCs requires the interaction with its ligand CD40L on T cells, particularly important for Tfh cells, to exert their stimulatory properties, including memory B cell development, germinal center formation or macrophage activation with induction of proinflammatory cytokines and reactive oxygen and nitrogen species, among others (Elgueta et al., 2009; Laman et al., 2017). Thus, both ICOS and CD40/CD40L play a significant role in Tfh cells differentiation and proliferation, which has been associated with a reduction of Th1-driven immunopathology, the support of CD8^+^ T cell response, and a more robust antibody response (Vella et al., 2017). In our study, *ICOS* showed a significant up-regulation at the end of the study in the lung from both infected groups, whereas *CD40* expression was already enhanced from 6 dpi. These results suggest that the activation of both *ICOS* and CD40 along PRRSV infection may play a dual role, on one hand, inducing macrophage activation to a proinflammatory status, and on the other hand, giving place to a microenvironment which favor Tfh cells differentiation.

Finally, it is described that immune checkpoints may mediate the immunomodulatory functions of Tregs during the development and progression of several infectious diseases (Sun et al., 2021). Noteworthy, *FOXP3,* the transcription factor that masters the differentiation and function of Tregs, was found to be upregulated at mRNA and protein level in the lung and tracheobronchial lymph node from Lena and 3249-infected piglets in a parallel studio, and it was associated with the constraint and recovery of lung injury during acute PRRSV infection (Sánchez-Carvajal et al., 2020; Ruedas-Torres et al., 2021a). According with these findings, a significant correlation between *FOXP3* and most of the immune checkpoint’s molecules were found in the tracheobronchial lymph node in both infected groups, but in the case of virulent Lena strain, the majority of these coinhibitory molecules were also correlated with *FOXP3* at lung level. These findings could suggest a migration of induced Tregs from the regional lymph node to the lung in order to mitigate the high inflammatory response occurring in the lung, particularly in the case of Lena-infected piglets.

Altogether our results highlight a mild increase of costimulatory molecules together with an earlier and stronger up-regulation of coinhibitory molecules in target organs from PRRSV-1-infected animals, especially in the lung from virulent Lena-infected animals. Simultaneous expression of coinhibitory immune checkpoints evidenced by the strong correlation found among them, suggests a synergistic effect of these molecules, most probably addressed to control the exacerbated inflammatory response and limit associated tissue injury. Further studies, specially focus on T-cell function and cytokine production, should be addressed to determine the role of these molecules in later stages of PRRSV infection.

## Data availability statement

The raw data supporting the conclusions of this article will be made available by the authors, without undue reservation.

## Ethics statement

The animal study was reviewed and approved by IRTA Ethics Committee and by the Catalan Autonomous Government (Project 3,647; FUE-2017-00533413) and carried out following the European Union guidelines (Directive 2010/63/EU).

## Author contributions

JG-L, IR-G, and LC conceived, designed, and performed the project. FP, IR-G and JG-L helped in the animal experiments and sample collection. JS-C, IR-T, and FL-M made the laboratory experiments and analyzed the data. IR-T and JS-C wrote the manuscript. IR-G and JG-L reviewed the manuscript. LC, FP, and JG-L supervised the study and contributed to reagents, materials, and analysis tools. All authors contributed to the article and approved the submitted version.

## Funding

JS-C is supported by a “Margarita salas” contract of the Spanish Ministry of Universities. This work was supported by the Spanish Ministry of Economy and Competitiveness (#AGL2016-76111-R and PID2019-109718GB-I00).

## Conflict of interest

The authors declare that the research was conducted in the absence of any commercial or financial relationships that could be construed as a potential conflict of interest.

## Publisher’s note

All claims expressed in this article are solely those of the authors and do not necessarily represent those of their affiliated organizations, or those of the publisher, the editors and the reviewers. Any product that may be evaluated in this article, or claim that may be made by its manufacturer, is not guaranteed or endorsed by the publisher.

## Footnotes

^1^ www.qbaseplus.com

^2^ www.primer3plus.com
